# Long non-coding RNA CCAT1 modulates neuropathic pain progression through sponging miR-155

**DOI:** 10.18632/oncotarget.21192

**Published:** 2017-09-23

**Authors:** Lidong Dou, Hongqi Lin, Kaiwei Wang, Guosong Zhu, Xuli Zou, Enqiang Chang, Yongfeng Zhu

**Affiliations:** ^1^ Department of Anesthesiology, Henan Provincial People's Hospital, Zhengzhou, Henan 450003, China

**Keywords:** neuropathic pain, long non-coding RNAs, lncRNAs, CCAT1, miR-155

## Abstract

Neuropathic pain is caused by dysfunction or primary injury of the somatosensory nervous system. Long noncoding RNAs (lncRNAs) play important roles in the development of neuropathic pain. However, the effects of lncRNA colon cancer associated transcript-1 (CCAT1) in neuropathic pain have not been reported. The model of bilateral sciatic nerve chronic constriction injuries (bCCI) is regarded as long-lasting mechanical hypersensitivity and cold allodynia, which is the representative symptom in the human subjects suffering from the neuropathic pain. In this study, we found that CCAT1 expression was decreased in the spinal dorsal horn, dorsal root ganglion (DRG), hippocampus, and anterior cingulate cortex (ACC) of rats with bCCI. The rats of bCCI presented the cold allodynia after the 14^th^ day of postoperation. We furtherly showed that lncRNA CCAT1 decreased miR-155 expression and enhanced Serum and glucocorticoid regulated protein kinase 3 (SGK3) expression in the NGF-differentiated PC12 cell. We found that miR-155 expression was increased in the spinal dorsal horn, DRG, hippocampus, and ACC of rats with bCCI injuries. However, SGK3 expression was downregulated in the spinal dorsal horn, DRG, hippocampus, and ACC of rats with bCCI injuries. Moreover, lncRNA CCAT1 overexpression could alleviate the pain thresholds and inhibited expression of SGK3 could rescue this effect. In conclusion, these results suggested the crucial roles of CCAT1 and SGK3 in the neuropathic pain.

## INTRODUCTION

Neuropathic pain is one kind of indirect or direct pain caused by the dysfunction or primary injury of the somatosensory nervous system, and is considered as one of the most serious public health problems [1–4]. It is difficult to treat effectively for the majority of neuropathic pain since all current therapies only alleviate the symptoms rather than curing or addressing the problem [5–8]. The main causes are that the molecular mechanisms underlying the neuropathic pain development remain elusive [9–11]. Thus, it is important to study the molecular mechanisms of neuropathic pain development.

Long non-coding RNAs (lncRNAs) are longer than 200 nucleotides with no protein-coding or limited capacity [12–15]. Increasing studies have suggested that lncRNAs can server crucial roles in cell development, proliferation, differentiation, migration and invasion [16–20]. Recent evidences have demonstrated that lncRNAs are upregulated or downregulated in neuropathic pain models, which support the potential role of lncRNAs as a novel group of targets for the treatment of neuropathic pain [21–23]. Colon cancer associated transcript-1 (CCAT1) was a novel lncRNA which was demonstrated to be upregulated in the colon cancer and gastric cancer [24]. LncRNA CCAT1 plays important roles in the proliferation, migration and invasion. However, the role of CCAT1 was still uncoverd in the developmen of neuropathic pain.

In our study, we found that CCAT1 expression was decreased in the spinal dorsal horn, DRG, hippocampus, and ACC of rats with bCCI injuries. LncRNA CCAT1 overexpression could alleviate the pain thresholds partly through regulating miR-155/SGK3 expression.

## RESULT

### Mechanical hypersensitivity and acetone tests

**Figure 1:**
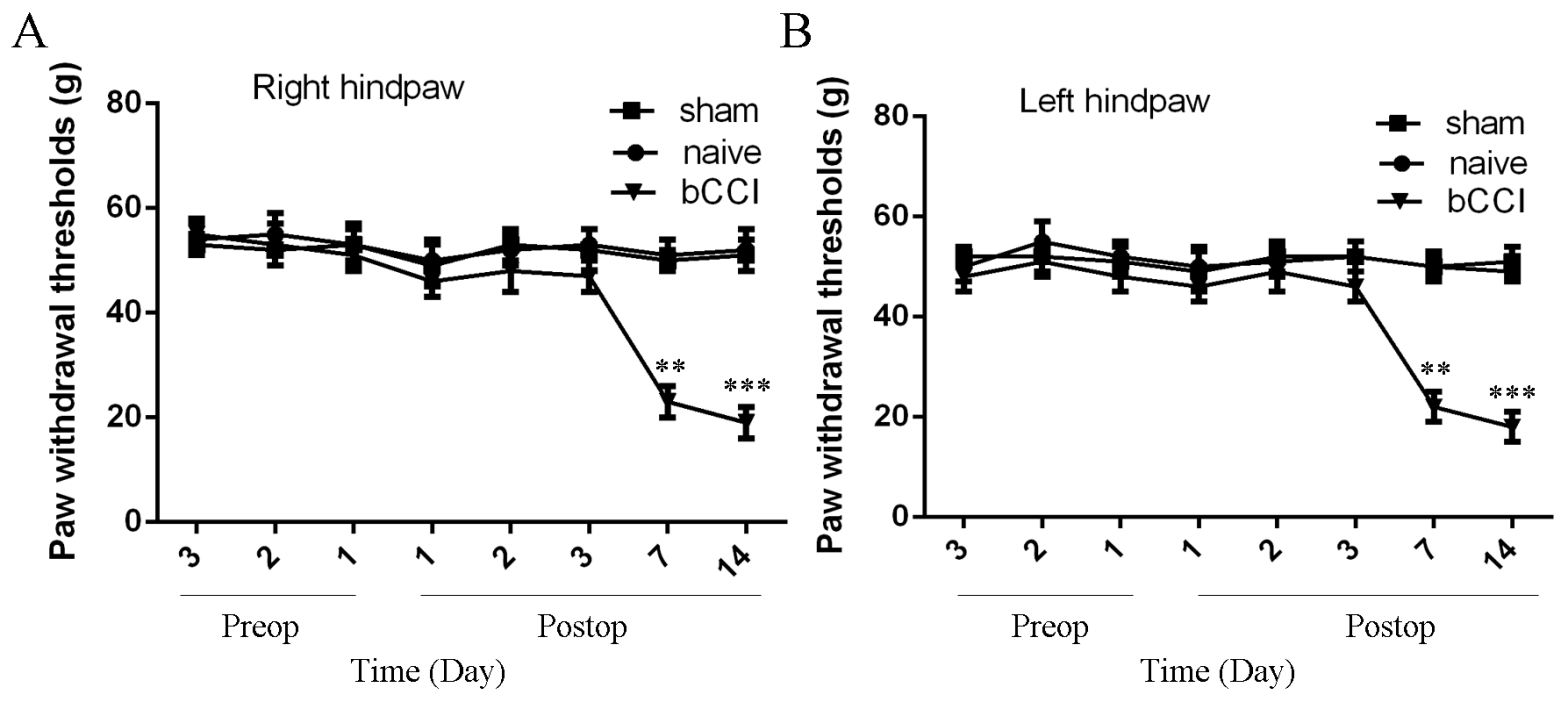
Mechanical sensitivity threshold of the model rats **(A)** Left hindpaw; **(B)** Right hindpaw. Rats submitted to sciatic ligation developed tactile stimulus-induced hypersensitivity at 7^th^ and 14th day postsurgery, whereas sham-operated and naive rats had no change in their sensitivity. ^**^p<0.01 and ^***^p<0.001.

**Figure 2:**
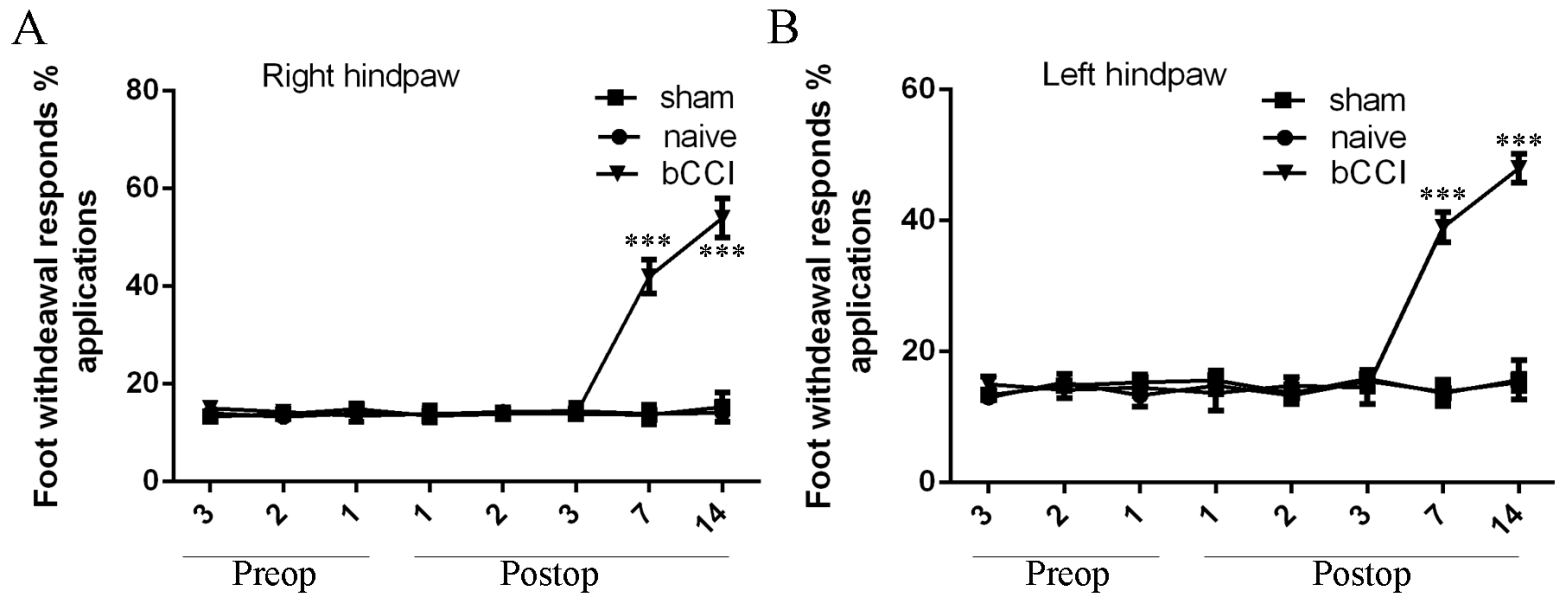
Acetone tests of the model rats **(A)** Left hindpaw; **(B)** Right hindpaw. Rats submitted to sciatic ligation developed cold allodynia at 7th and 14th day postsurgery, whereas sham-operated and naive rats showed no change in cold sensitivity. ^***^p<0.001.

We firstly detected the mechanical sensitivity threshold of the model rats. We demonstrated that the mechanical sensitivity threshold of bCCI group rats was significantly lower on the postoperative day 7 and 14 than in the sham-operated and nave group rats both in the right and left hindpaw (Figure 1A and 1B). In addition, we also found that cold allodynia of the bCCI group rats was significantly lower on the postoperative day 7 and 14 than in the sham-operated and nave group rats both in the right and left hindpaw (Figure 2A and 2B).

### CCAT1 expression was downregulated in the bCCI model

**Figure 3:**
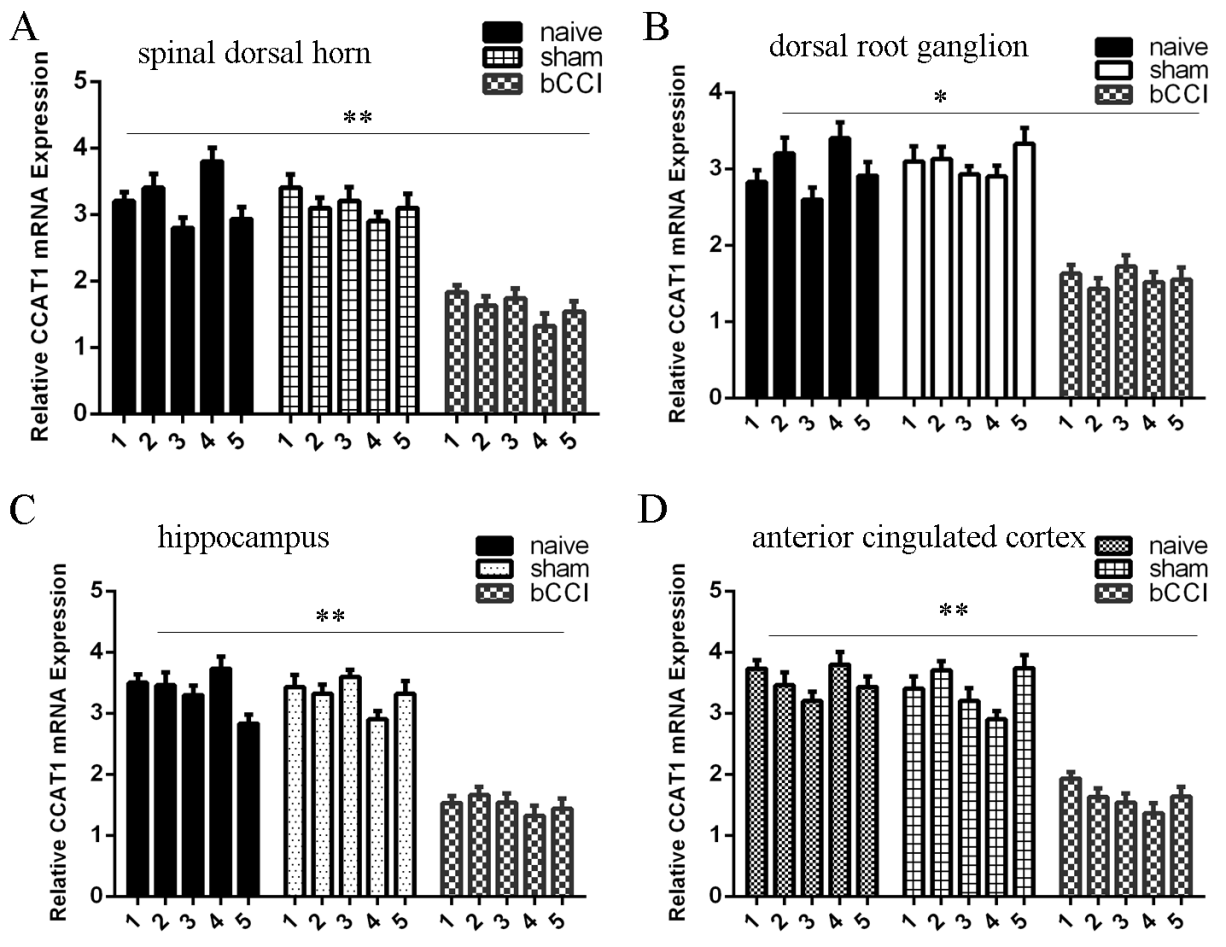
CCAT1 expression was downregulated in the bCCI model **(A)** The expression of CCAT1 in the spinal dorsal horn was determined by qRT-PCR. U6 was used as the internal control. **(B)** The expression of CCAT1 was also lower in the dorsal root ganglion (DRG) than in the sham-operated and nave group rats. **(C)** The expression of CCAT1 in the hippocampus was determined by qRT-PCR. **(D)** The CCAT1 expression in the ACC was determined by qRT-PCR. ^*^p<0.05 and ^**^p<0.01.

Next, we determined CCAT1 expression in the different regions of the rat nervous system. We demonstrated that CCAT1 expression level was downregulated in the spinal dorsal horn of bCCI rats compared to the sham-operated and nave group rats (Figure 3A). In addition, CCAT1 expression was also lower in the DRG (Figure 3B), hippocampus (Figure 3C), and ACC (Figure 3D) than in the sham-operated and nave group rats.

### CCAT1 suppressed miR-155 expression in the PC12 cell

**Figure 4:**
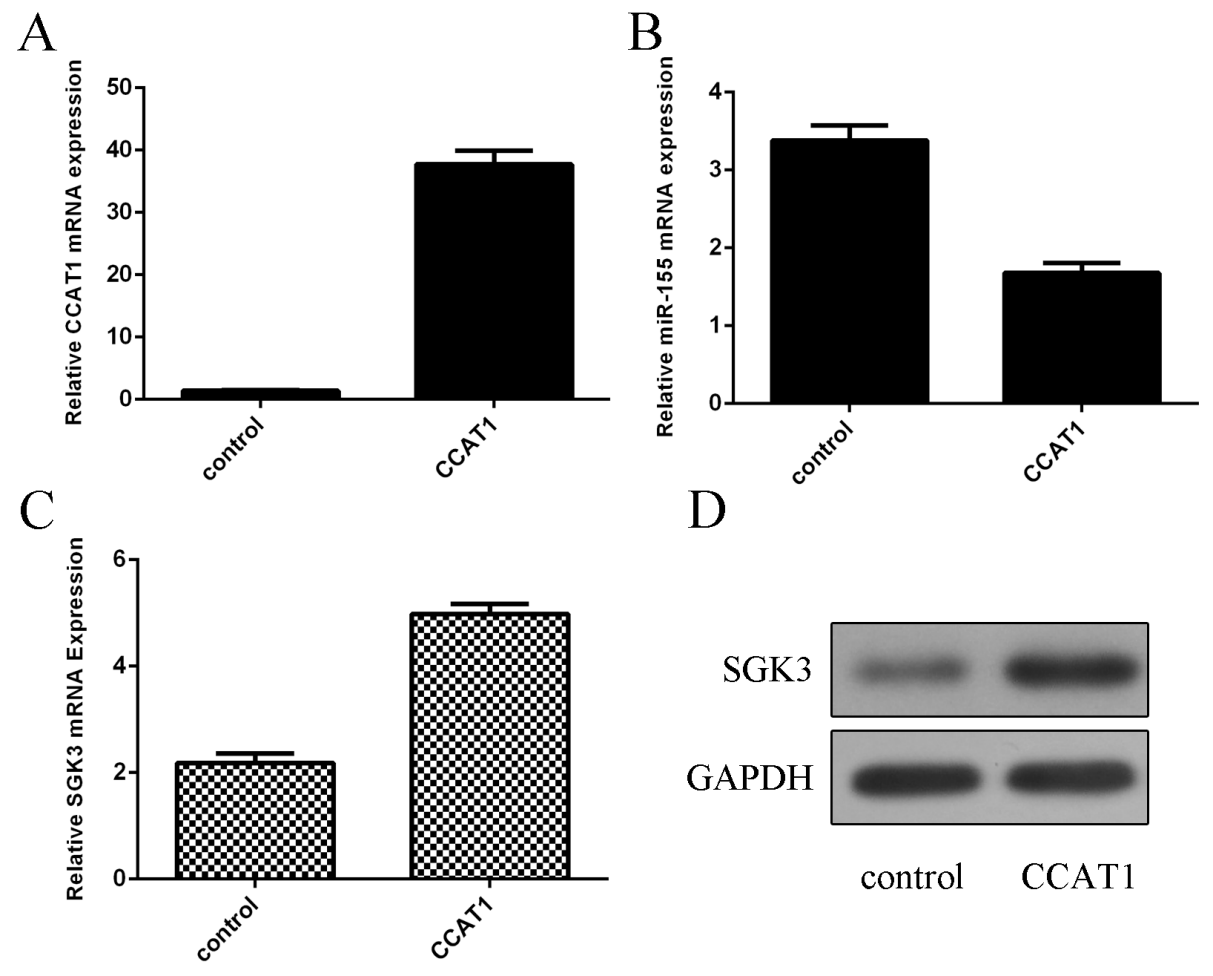
CCAT1 suppressed the miR-155 expression in the PC12 cell **(A)** The expression of CCAT1 was detected in the PC12 cell which treated with pCDNA-CCAT1 by qRT-PCR. **(B)** Elevated expression of CCAT1 suppressed the miR-155 expression in the PC12 cell. **(C)** Ectopic expression of CCAT1 enhanced the SGK3 expression in the PC12 cell. **(D)** The protein expression of SGK3 was measured by Western blot. GAPDH was used as the internal control.

We showed that CCAT1 expression was significantly upregulated in the PC12 cell after treated with pcDNA-CCAT1 (Figure 4A). Ectopic expression of CCAT1 decreased miR-155 expression in the PC12 cell (Figure 4B). Moreover, overexpression of CCAT1 increased SGK3 expression, which was the direct target gene of miR-155 (Figure 4C and 4D).

### MiR-155 expression was upregulated in the bCCI model

**Figure 5:**
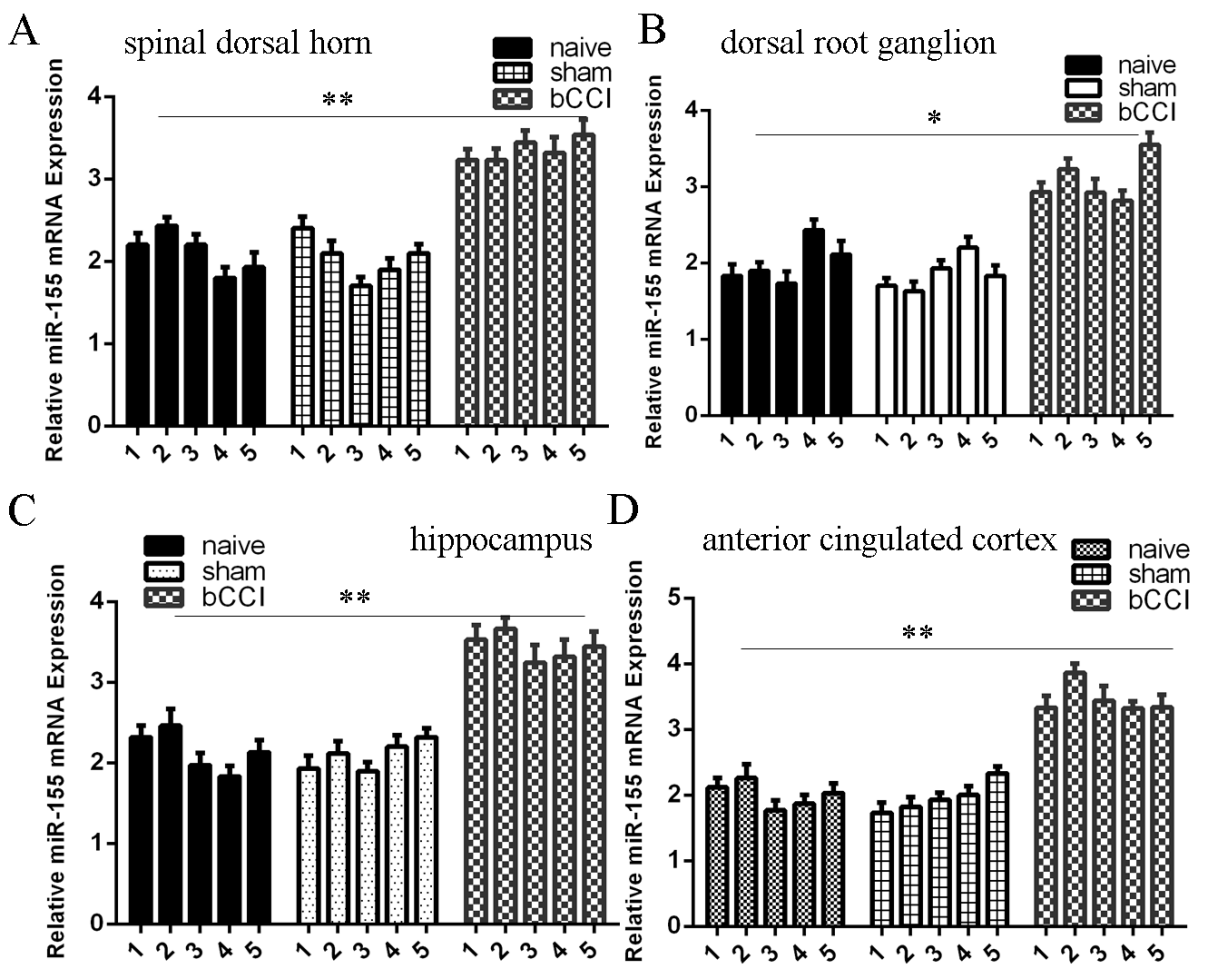
miR-155 expression was upregulated in the bCCI model **(A)** The expression of miR-155 in the spinal dorsal horn was determined by qRT-PCR. U6 was used as the internal control. **(B)** The expression of miR-155 was also lower in the dorsal root ganglion (DRG) than in the sham-operated and nave group rats. **(C)** The expression of miR-155 in the hippocampus was determined by qRT-PCR. **(D)** The miR-155 expression in the ACC was determined by qRT-PCR. ^*^p<0.05 and ^**^p<0.01.

Next, we determined miR-155 and SGK3 expression in the different regions of the rat nervous system. We found that miR-155 expression level was upregulated in the spinal dorsal horn (Figure 5A), DRG (Figure 5B), hippocampus (Figure 5C), and ACC (Figure 5D) compared to sham-operated and nave group rats.

### SGK3 expression was downregulated in the bCCI model

**Figure 6:**
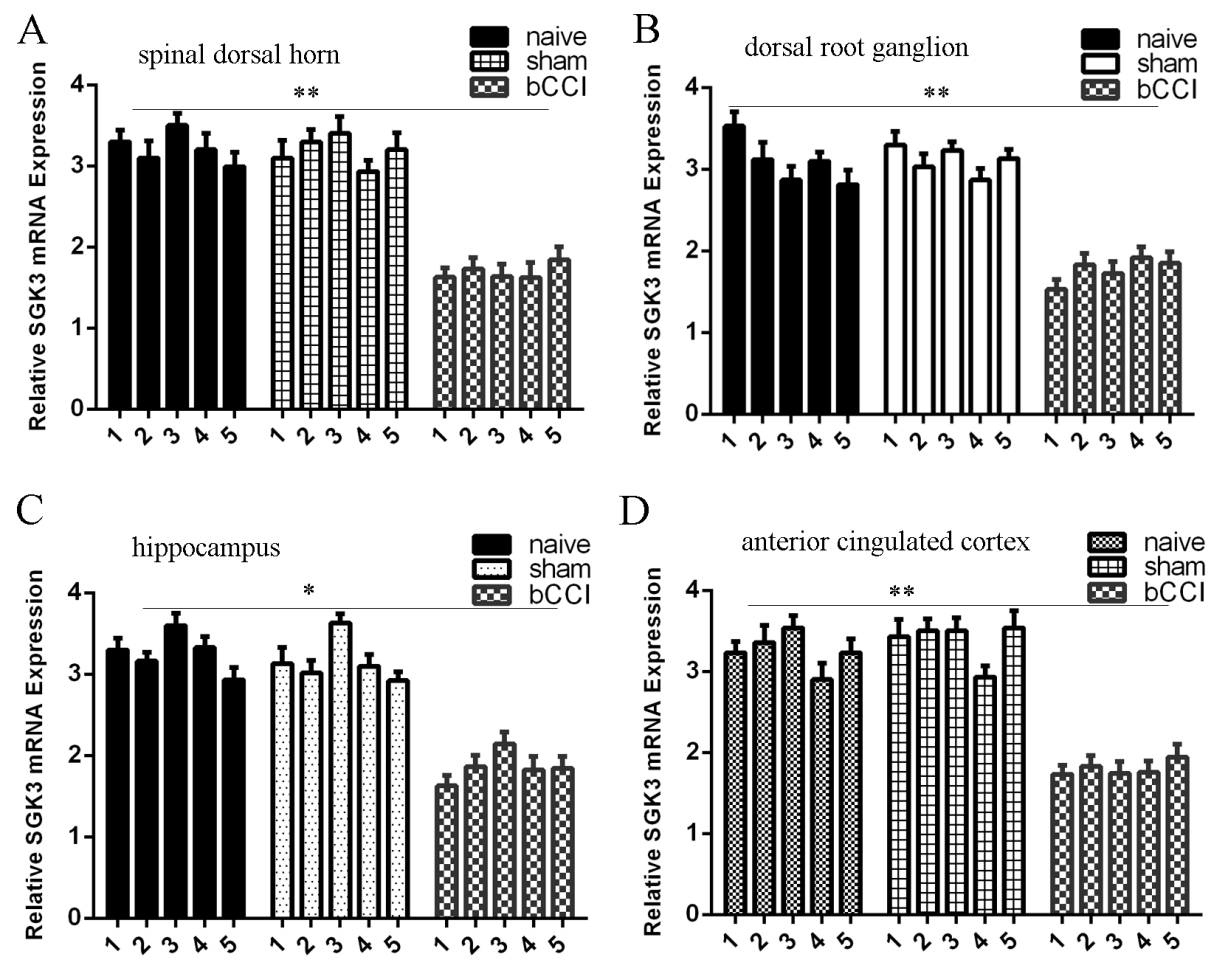
SGK3 expression was downregulated in the bCCI model **(A)** The expression of SGK3 in the spinal dorsal horn was determined by qRT-PCR. GAPDH was used as the internal control. **(B)** The expression of SGK3 was also lower in the dorsal root ganglion (DRG) than in the sham-operated and nave group rats. **(C)** The expression of SGK3 in the hippocampus was determined by qRT-PCR. **(D)** The SGK3 expression in the ACC was determined by qRT-PCR. ^*^p<0.05 and ^**^p<0.01.

Furthermore, we measured SGK3 expression in different regions of the rat nervous system in different groups. We showed that SGK3 expression was downregulated in the spinal dorsal horn (Figure 6A), DRG (Figure 6B), hippocampus (Figure 6C), and ACC (Figure 6D) compared to sham-operated and nave group rats.

### Functional analysis of CCAT1 in the neuropathic pain model

**Figure 7:**
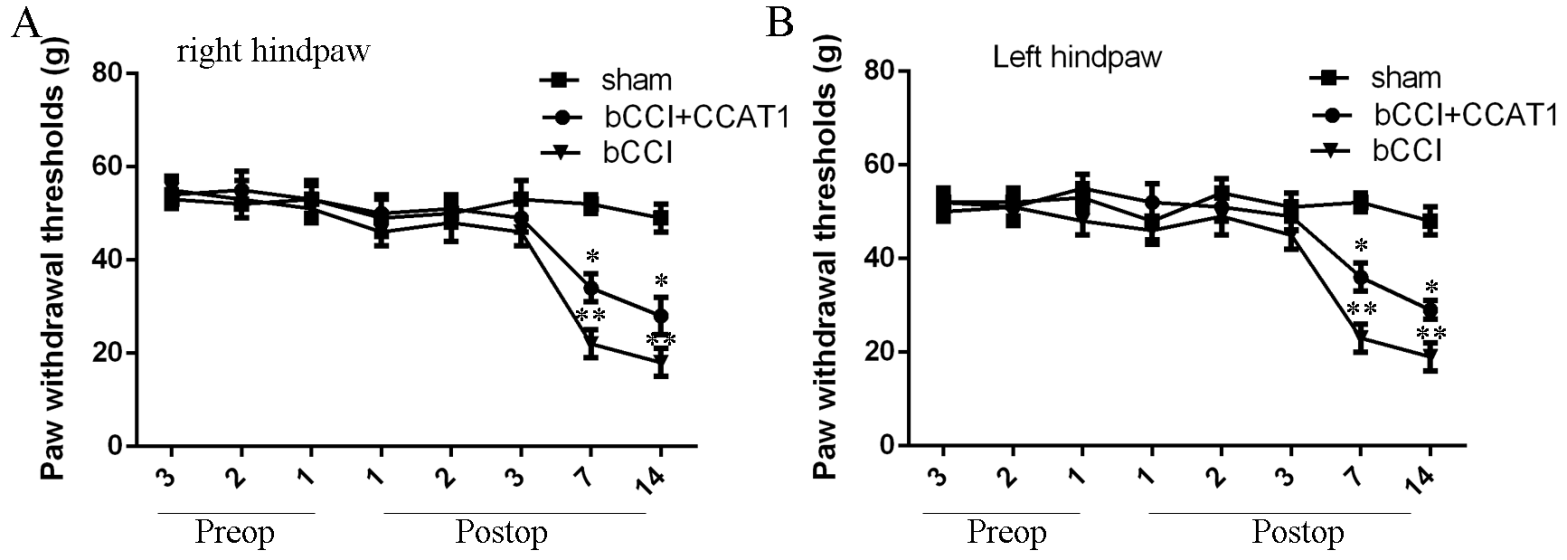
Functional analysis of CCAT1 in neuropathic pain model **(A)** CCAT1 overexpression could improve the pain threshold for bCCI rats in the right hindpaw. **(B)** CCAT1 overexpression could improve the pain threshold for bCCI rats in the left hindpaw. ^*^p<0.05 and ^**^p<0.01.

In order to determine the functional role of CCAT1 in the pain model, pcDNA-CCAT1 vector was used in the pain model. We showed CCAT1 overexpression could improve the pain threshold and cold allodynia for bCCI rats, indicating that overexpression of CCAT1 could lighten the pain threshold and cold allodynia for model rats (Figure 7A and 7B).

### Functional analysis of CCAT1/SGK3 in neuropathic pain model

**Figure 8:**
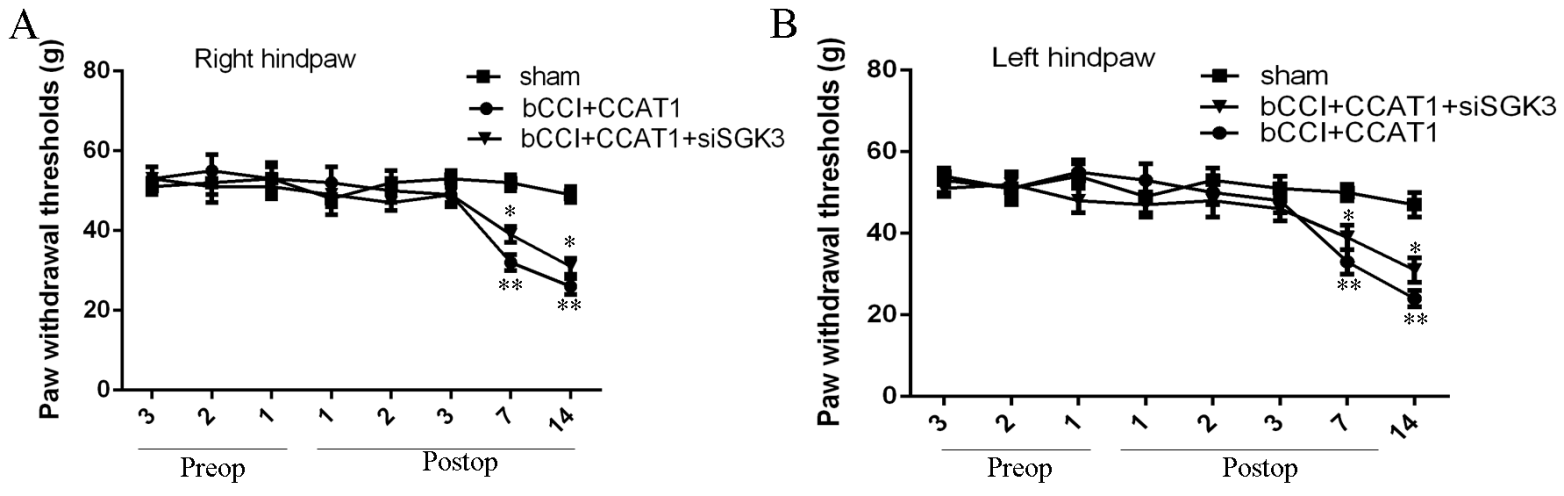
Functional analysis of CCAT1/SGK3 in neuropathic pain model **(A)** Compared to the controls, inhibition of SGK3 expression can decrease the pain threshold effect of CCAT1 overexpression treated bCCI rats in the right hindpaw. **(B)** Compared to the controls, inhibition of SGK3 expression can decrease the pain threshold effect of CCAT1 overexpression treated bCCI rats in the left hindpaw. ^*^p<0.05 and ^**^p<0.01.

To determined the role of SGK3 in the mechanism of CCAT1 in neuropathic pain model. si-SGK was transfected into the neuropathic pain model. Compared to the controls, inhibition of SGK3 expression decreased the pain threshold and cold allodynia effect of CCAT1 overexpression treated bCCI rats, indicating that CCAT1 could lighten pain threshold and cold allodynia through enhancing SGK3 expression (Figure 8A and 8B).

## DISCUSSION

Neuropathic pain is a common public health problem and is caused by dysfunction or injury of somatosensory nervous system [25–27]. The effects of lncRNA CCAT1 in neuropathic pain have not been reported. The model of bCCI is regarded as long-lasting mechanical hypersensitivity and cold allodynia, which is representative symptom in the human subjects suffering from the neuropathic pain. In our study, we found that CCAT1 expression was decreased in the spinal dorsal horn, DRG, hippocampus, and ACC of rats with bCCI injuries. The rats of bCCI presented the cold allodynia after the 14^th^ day of postoperation. We showed that lncRNA CCAT1 decresaed the expression of miR-155 and enhanced the SGK3 expression in the NGF-differentiated PC12 cell. We found that miR-155 expression was increased in the spinal dorsal horn, DRG, hippocampus, and ACC of rats with bCCI injuries. However, SGK3 expression was downregulated in the spinal dorsal horn, DRG, hippocampus, and ACC of rats with bCCI injuries. Moreover, lncRNA CCAT1 overexpression could alleviate the pain thresholds and inhibited expression of SGK3 could rescue this effect. These results suggested the crucial roles of CCAT1 and SGK3 in the neuropathic pain.

Previous studies suggested that lncRNAs played important roles in the development of neuropathic pain [2, 22, 28, 29]. For example, Wang et al [30]. showed LncRNA uc.48+ was involved in the diabetic neuropathic pain regulated by the P2×3 receptor in dorsal root ganglia. Peng et al [28]. showed that lncRNA NONRATT021972 expression was upregulated in the Type 2 diabetes mellitus (T2DM) rat model. The expression of NONRATT021972 in the T2DM patient serum was also upregulated compared to the healthy subjects. Inhibition of NONRATT021972 can decrease the hyperalgesia potentiated by the TNF-α in T2DM rats. Liu et al [31]. demonstrated that the expression of NONRATT021972 was upregulated in the DRG of diabetes mellitus (DM) rats and NONRATT021972 siRNA could promoted the thermal withdrawal latency, the sensory nerve conduction velocity and mechanical withdrawal threshold of rat tail nerves. In our study, we indicated that lncRNA CCAT1 expression was decreased in the spinal dorsal horn, DRG, hippocampus, and ACC of rats with bCCI injuries. Moreover, lncRNA CCAT1 overexpression could alleviate the pain thresholds.

Previous study indicated that miR-155 expression was upregulated in the neuropathic pain rats compared to the sham and control group and inhibition of miR-155 decreased the pain thresholds [32]. Serum and glucocorticoid regulated protein kinase 3 (SGK3) was indentified as the direct target gene of miR-155. The expression of SGK3 was negatively related with miR-155 expression. Overexpression of SGK3 could decrease the pain thresholds. Previous studies demonstrated that lncRNAs could act as a ceRNA to inhibit miRNA expression [13, 33, 34]. In our study, we found that overexpression of CCAT1 could inhibit miR-155 expression and enhance the SGK3 expression, which was a direct target gene of miR-155. Furthermore, we indicated that the expression of miR-155 was increased in the spinal dorsal horn, DRG, hippocampus, and ACC of rats with bCCI injuries. However, SGK3 expression was downregulated in the spinal dorsal horn, DRG, hippocampus, and ACC of rats with bCCI injuries. In addition, we showed that lncRNA CCAT1 overexpression could alleviate the pain thresholds and inhibited expression of SGK3 could rescue this effect.

In conclusion, our data revealed that lncRNA CCAT1 expression was significantly decreased in the spinal dorsal horn DRG, hippocampus, and ACC of bCCI rats. LncRNA CCAT1 overexpression could alleviate the pain thresholds partly through regulating SGK3 expression. These results suggested that lncRNA CCAT1 played crucial roles in the development of neuropathic pain.

## MATERIALS AND METHODS

### Neuropathic pain model construction

Our study were approved by the Animal Care and Committee of Henan Provincial People's Hospital and used in the accordance with the guideline of International Association for the Study of Pain. Thirty-five clean adult female Spragure-Dawley (SD) rats were randomly divided into three groups (bCCI, sham, and naive) and hosed at the climate-controlled room under a 12/12 hours light/dark cycle with cycle with free access to water and food. Bilateral chronic constriction injury (bCCI) model was created for neuropathic pain as previous report [25]. Rat was anesthetized using sodium pentobarbital through intraperitoneal Injection. The sciatic nerve was exposed at the level of the middle of the thigh by blunt dissection through biceps femoris. Each sciatic nerve was identified the trifurcation and then freed from surrounding loose connective tissues before the 4 snug ligatures of four-zero chromic gut suture was placed around them. The sciatic nerves of Sham-operated rats were exposed but not ligated and the naïve rats were not operated upon.

### Mechanical withdrawal test

Electronic von Frey filament (IITC Life Sciences, Woodland Hills, CA, USA) was used to determine the mechanical hyperalgesia. Rat was acclimated to the mesh bottom cages for five to fifteen minutes. Pressure was applied to the plantar surface of each hindpaw of rat through the mesh floor from below with the electronic Von Frey filament. Each hindpaws was alternated with this process for a total of 10 times each day (5 per hindpaw). The force used at the time of paw withdrawal was marked. Pretesting was performed for three consecutive days before bilateral constriction injury surgery. The threshold for the paw withdrawal in response to mechanical stimuli was determined at the 1, 2 and 3 days before surgery and 1,2,3 and 7 and 14 days after surgery. The L4-6 dorsal horn, bilateral L4-6 DRG of spinal cord segments, anterior cingulated cortex (ACC) and hippocampus were harvested and then frozen at the liquid nitrogen.

### Acetone test

Cold allodynia was measured on the three consecutive days before surgery and on 1, 2, 3 and 7 and 14 day after surgery. A drop of acetone was applied to each hindpaw through the polyethylene plastic tubing on the room temperature. A withdrawal of hindpaw in response to spread of the acetone over the surface of hindpaw was meann as a sign of the cold allodynia. Data was regarded as the percentage of the applications that induced the hindpaw withdrawal response. An augment in percentage of application eliciting the withdrawal response compared to the control was regarded as the development of promoted cold sensitivity.

### Cell culture

PC12 cell (pheochromocytoma cell lines of rats) was collected from the cell bank center of the of Institute of Basic Medical Sciences, Chinese Academy of Medical Sciences (Beijing, China) and was cultured in the RPMI-1640 media (Gibco; Invitrogen; Life Technologies, Germany) supplemented with donor horse serum and fetal bovine serum. NGF-2.5 (50 ng/mL, Peprotech) was added to the medium and maintained for seven to ten days.

### Quantitative real-time RT-PCR

Total RNA from cell or tissue was extracted with the TRIzol Reagent (Invitrogen, CA, USA). Real-time PCR was performed to determine the CCAT1 and miR-155 and mRNA expression by using the SYBRW Green PCR Master Mix (Roche, Germany) on the LightCycler 480 system (Roche, Basel, Switzerland). The relative expression level of XX was normalized to GAPDH. U6 was performed as the internal control. The result was measured following to the comparative 2^-DDCT^ method.

### Western blot

Primary antibodies were used in our study including xx (Bioworld Technology, Louis Park, MN, USA). Equal amount of the protein was separated with 12% SDS-PAGE and then transferred to PVDFmembrane (Beyotime). The membrane was blocked in nonfat dry milk for 1 hour and incubated with primary antibodies overnight. After washed three times in TBST, the membrane was incubated with HRP-linked secondary antibodies for 1 hour. The signal was measured by using ECL detection system. GAPDH was used as the loading control.

### Statistical analysis

Result was presented as mean ± SD (standard deviation). Statistical difference was analyzed by using SPSS17.0 software (IBM, Chicago, IL, USA). Difference between each group was measured by unpaired two-tailed Student t-test. P-value <0.05 was considered statistically significant.
